# Training attention control of very preterm infants: protocol for a feasibility study of the Attention Control Training (ACT)

**DOI:** 10.1186/s40814-020-0556-9

**Published:** 2020-02-10

**Authors:** Oliver Perra, Sam Wass, Alison McNulty, David Sweet, Kostas Papageorgiou, Matthew Johnston, Aaron Patterson, Delfina Bilello, Fiona Alderdice

**Affiliations:** 1 School of Nursing and Midwifery, Queen’s University Belfast, Medical Biology Building, 97 Lisburn Road, Belfast, BT9 7BL Northern Ireland, UK; 2 Centre for Evidence and Social Innovation, Queen’s University Belfast, Belfast, Northern Ireland, UK; 3 School of Psychology, University of East London, London, UK; 4 TinyLife, The Premature Baby Charity for Northern Ireland, Belfast, UK; 5 Health and Social Care Belfast Trust, Belfast, Northern Ireland, UK; 6 School of Psychology, Queen’s University Belfast, Belfast, Northern Ireland, UK; 7 Nuffield Department of Population Health, University of Oxford, Oxford, UK

**Keywords:** Infant, Premature, Feasibility study, Attention, Computerized Cognitive Training, Eye-tracking methodology

## Abstract

**Background:**

Children born preterm may display cognitive, learning, and behaviour difficulties as they grow up. In particular, very premature birth (gestation age between 28 and less than 32 weeks) may put infants at increased risk of intellectual deficits and attention deficit disorder. Evidence suggests that the basis of these problems may lie in difficulties in the development of executive functions. One of the earliest executive functions to emerge around 1 year of age is the ability to control attention. An eye-tracking-based cognitive training programme to support this emerging ability, the Attention Control Training (ACT), has been developed and tested with typically developing infants. The aim of this study is to investigate the feasibility of using the ACT with healthy very preterm (VP) infants when they are 12 months of age (corrected age). The ACT has the potential to address the need for supporting emerging cognitive abilities of VP infants with an early intervention, which may capitalise on infants’ neural plasticity.

**Methods/design:**

The feasibility study is designed to investigate whether it is possible to recruit and retain VP infants and their families in a randomised trial that compares attention and social attention of trained infants against those that are exposed to a control procedure. Feasibility issues include the referral/recruitment pathway, attendance, and engagement with testing and training sessions, completion of tasks, retention in the study, acceptability of outcome measures, quality of data collected (particularly, eye-tracking data). The results of the study will inform the development of a larger randomised trial.

**Discussion:**

Several lines of evidence emphasise the need to support emerging cognitive and learning abilities of preterm infants using early interventions. However, early interventions with preterm infants, and particularly very preterm ones, face difficulties in recruiting and retaining participants. These problems are also augmented by the health vulnerability of this population. This feasibility study will provide the basis for informing the implementation of an early cognitive intervention for very preterm infants.

**Trial registration:**

Registered Registration ID: NCT03896490. Retrospectively registered at Clinical Trials Protocol Registration and Results System (clinicaltrials.gov).

## Background

The number of surviving children born premature has increased across many countries around the world [9, 10]. Advances in obstetric care since the early 1990s have also contributed to diminishing risks for disability of children born preterm, e.g. reduction in the prevalence of cerebral palsy [61]. Nonetheless, premature birth is still associated with increased risk for intellectual deficits [15, 16, 48, 67] and poorer school attainment [4]. Several studies suggest that this risk increases with lower gestational age [65]. In particular, newborns born very premature (VP), i.e. those born between 28 and less than 32 weeks gestation age, are at increased risk of significant intellectual deficits [8, 16, 30, 33, 40, 45, 60], learning difficulties [2, 39], attention problems [2, 23, 64], problem behaviours [6, 12, 19, 42], and developmental disorders such as attention deficit with hyperactivity disorder (ADHD) [13, 14, 26, 28, 46].

Several studies have suggested that the bases of these problems may lie in anomalies in the way children born VP regulate and control the acquisition of information [37, 38, 41]. These problems may be evident from an early age. For example, Rose and colleagues [52, 53, 55, 56] conducted several studies on a cohort of preterm infants weighing less than 1750 g at birth and with average gestational age 29 weeks: they reported that preterm infants displayed less efficient and more immature patterns of attention in standardised tasks that required effortful processing of stimuli rather than just orienting responses. Downes and colleagues [21] showed that 1-year-old VP infants were less capable of allocating sufficient attention to targets during a visual attention task. Sun and others [64] also reported that at 8 months (corrected age), VP infants displayed more significant problems in inhibiting attention to irrelevant information or distracters compared with full-term controls during an A-not-B task. Collectively, these findings suggest that VP infants display problems allocating and controlling attention according to task demands.

VP infants’ difficulties in attention control may represent early signs of deficits in processes that are fundamental to the development of learning skills. Attention control refers to the ability to select actively what to pay attention to and what to ignore. This ability starts to emerge by 1 year of age in typically developing infants [18, 20]. The emergence of attention control represents a shift from exogenously controlled attention systems whereby responses are determined purely by the external environment towards an increased role for endogenous and volitional processes in which responses are determined by factors intrinsic to the person attending. With increasing age, attention is deployed more flexibly in order to process task-relevant information and inhibit irrelevant information. Attention control is therefore considered to play a foundational role in the development of later-emerging executive functions (EFs), such as planning and cognitive flexibility [5, 32]. EFs are, in turn, thought to be important for facilitating learning and behaviour regulation, and anomalies in their development may impact on the development of other cognitive abilities [16]: the latter study indicated that EFs mediate the association between premature birth and poorer intellectual abilities of children born VP when compared with full-term children. Studies by Rose et al. also suggested that EFs can explain the association between prematurity and lower educational attainment of children born preterm [57, 58]. In conclusion, early-appearing problems in the control of attention in children born very preterm may generate a cascade of effects, which involve deficits in cognitive flexibility in toddlerhood, poorer EFs [72], and ultimately deficits in cognitive abilities and lower educational attainment.

According to this developmental view, early interventions play a key role in targeting and addressing problems and deficits in foundational abilities, such as attention control. Indeed, evidence from systematic reviews suggests cognitive training programmes produce greater effects when delivered at younger ages [73]. The reason for this may lie in the greater plasticity of neural networks that control cognitive skills: intervening early may help support the development of key skills before deficits become entrenched [73]. Early interventions targeted at key early-emerging skills can provide at-risk infants with the necessary building blocks for later attainments [11, 34, 72].

A new intervention to target infants’ attention control has been developed, dubbed the Attention Control Training (ACT) [7, 71]. This intervention uses computer interfaces that monitor infants’ direction of gaze through eye-tracking: this allows the computer to generate interactive presentations whereby visual stimuli on the screen change and vary in response to infants’ gaze direction. Interactive presentations provide motivating rewards (e.g. animations with sounds) when infants control attention in ways that fulfil the varying demands of the task at hand. For example, in some tasks, infants receive the reward if they maintain attention on an object, suppressing the tendency to look at distracters that appear on the screen. In a different task set, infants have instead to scan a series of targets on the screen in order to detect a pre-specified target object. The intervention is innovative because it engages young infants using age-appropriate tasks. Furthermore, the adaptive procedures allow tailoring the training to each infant’s initial skill set.

The ACT has been trialled with typically developing children [71] and has provided evidence of transfer of effects to attention skills (e.g. sustained attention) [7, 71, 74], short-term memory [7, 71], and naturalistic social attention tasks [25], thus demonstrating generalizability and persisting effects in the short term [7, 71]. However, before the intervention can be tested with VP infants, a series of processes need to be investigated to underpin its implementation in a full-scale randomised trial. These processes are the subject of the study presented here.

### Aims of the study

Different types of early developmental intervention programmes exist that aim to improve cognitive abilities of preterm infants [62]. These interventions mostly focus on the home environment, parent-infant relationships, and parenting skills, but few focus on infant development alone. Computerised cognitive training programmes have been tested among pre- and school-age children who were born before term [1, 49], but not with younger preterm children. Furthermore, few studies have reported on the use of eye-tracking methods to examine preterm infants’ attention abilities [21, 36, 59, 66]. Therefore, more research is necessary to establish the feasibility of delivering a computerised cognitive training programme to VP infants, which relies on eye-tracking technology.

The study described here tests the feasibility of a randomised trial investigating the effectiveness of the ACT for infants born very preterm. The study aims to determine recruitment strategies for the population of interest; the process of randomisation; procedures to mask allocation status to the parents and the assessors; the acceptability of the intervention, and the baseline and outcome measures; participating infants’ completion of the training and baseline/outcome measures; quality of information collected from participating infants.

### Research questions

Primary question:

- Is the study feasible? Is it possible to recruit and retain families of infants born very preterm into the programme and collect baseline and outcome measures, over a period of five consecutive weeks when the child is 12 months (corrected age)? Recruitment and retention criteria used to assess the success of the feasibility study are stated in the statistical analyses section of this manuscript.

Secondary questions:

(b) Is the programme acceptable to participating parents and infants?
(c) Are the proposed baseline and outcome measures acceptable to participating parents and infants?
(d) Do VP infants engage with the training/control procedures?
(e) Do VP infants engage with the baseline/outcome tasks and do these measures provide reliable data?

## Methods

### Trial design

**Fig. 1:**
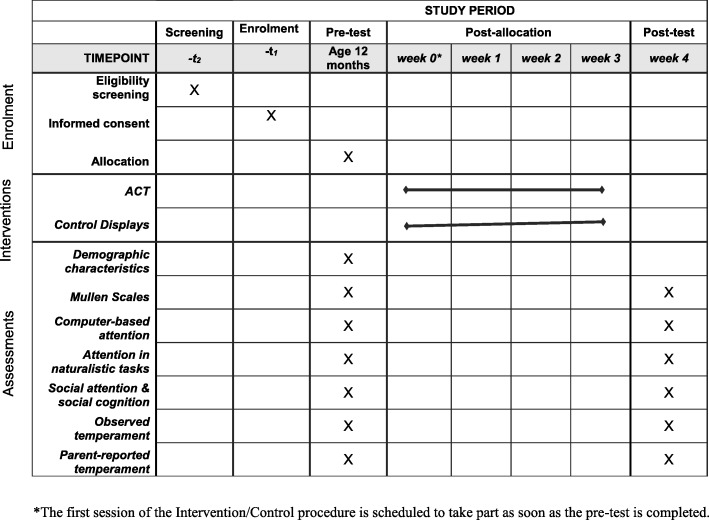
Schedule of enrolment, interventions, and assessments. *The first session of the intervention/control procedure is scheduled to take part as soon as the pre-test is completed

The study involves a feasibility randomised trial whereby eligible infants are allocated with 1:1 ratio to either the Attention Control Training (ACT) or a control procedure. Families and infants in both arms of the study are invited to take part in five sessions for five consecutive weeks, matching the protocol used in previous studies [31]. Researchers attempt to schedule the first session around the time the infant is 12 months (corrected age); however, inclusion criteria allow the infant to be between 11 and 13 months (corrected age) at the time of the first appointment. The first session involves a baseline assessment of attention and cognitive skills carried out by a blinded assessor (see “Blinding” section). If the infant is still in a calm and alert state, the infant also completes a training or control session within this first weekly session; however, if the infant is not calm and alert, the first training or control session is scheduled for the following weekly session. Study sessions in weeks 2, 3, and 4 involve the delivery of the training or the control procedure. In week 5 (post-test), infants complete the same battery of tasks used in week 1 (pre-test). To ensure that the experimenters administering the pre-post tests are blinded to the infant’s group allocation, separate researchers are used for the training/control visits (Experimenter 1) and for the pre-/post-test sessions (Experimenter 2). The infant’s group allocation (trained/control) is determined by opening a sealed envelope at the end of the pre-test session. In Fig. 1, we report the enrolment, interventions, and assessments schedule following the SPIRIT template.

The study protocol initially stipulated to run all the study sessions in a dedicated room in the premises of the local collaborating charity (Protocol version 12, dated 05 January 2018). However, after initial feedback from parents and discussion within the study steering group, we changed the protocol to allow parents to opt to conduct the training or control sessions in their own house (Protocol version 14, dated 16 November 2018). This change in the procedure was agreed in order to facilitate the participation of parents, by removing the need for weekly travel to the premises of the charity. The pre- and post-test sessions still take place in the dedicated room within the local charity, because the tasks administered during these sessions require more space and equipment (i.e. a table and cameras mounted on both sides of the room). To ensure that training or control sessions are comparable when delivered in a dedicated room in the charity premises and at home, the same set-up will be used in both situations. In particular, the participants will sit inside a cubic photo light tent, which avoids visual distractions and sudden changes in light conditions. Researchers will also strive to obtain parents’ and families’ collaboration in minimising any other potential source of distraction, e.g. loud or sudden noises. However, differences across the two settings will be monitored and investigated across the study.

### Participants

Eligibility criteria are infants born very preterm (gestational age 28 to less than 32 weeks); residing in Northern Ireland; age 12 months (+/−1 month) at the start of the study, corrected for prematurity. Exclusion criteria: Significant visual and/or hearing disabilities; congenital anomalies that may impact on their cognitive and sensory-motor development; a diagnosis of cerebral palsy; taking part in a trial (or have recently taken part in a trial) which may interfere with this study (e.g. by affecting concentration abilities or representing a significant burden for the family).

Participants are identified by collaborating neonatology practitioners in hospitals within the Belfast, South Eastern, and Northern Trust in Northern Ireland. Practitioners act as gatekeepers and ensure that information on the study is passed to families of infants that are eligible to take part and do not meet any exclusion criterion. Interested parents contact the research team to receive more details about the study and decide whether to take part or not. If parents agree to take part, the research team document their consent in writing and agree an appointment. Furthermore, the local charity for families of premature children passes the information on the study to eligible parents, who can decide to contact the research team if they are interested. Parents who receive information about the study from the charity are asked to consult with one of the collaborating neonatologists to ensure their child does not meet any exclusion criterion.

### Interventions

Infants in the ACT intervention watch interactive stimuli presentations which are contingent on infants’ direction of gaze. An eye-tracker records the infant’s eye movements in real-time. Interactive presentations provide animations in response to the infant’s gaze in ways that meet pre-specified criteria. We used training tasks to train abilities to search for a target among distracters (three games—‘Stars’, ‘Usual Suspects’, and ‘Disengagement’); short-term memory of objects embedded in scenes (‘Puzzle Memory’, ‘Windows’, ‘Tausendfuss’, and ‘Three Little Maids’); maintaining a goal (‘Butterfly’, and ‘FlyMe’). Further details on all of these tasks are provided in Additional file 2. In each session, tasks are evenly spread across the three categories. Researchers are instructed to attempt to present each game for at least 240 s without interruptions; however, training games are interrupted if the child becomes fussy or irritable, and fails to engage with the task for a continuous period of over 30 s. A suggested random order of the tasks is generated by the computer script in each training session. The researcher also assesses if the infant is still in calm and alert status before commencing any new task, and stops the sessions if the infant becomes restless or drowsy. A video camera mounted on the screen captures the infant’s face: this recording is used in order to check the quality of data produced and to complement potential loss of data.

The control procedure involves presenting cartoons on a screen while infants’ gaze direction is recorded using the same eye-tracker and camera. As with the training tasks, the infant is seated throughout on the parent’s lap. The crucial difference is that the stimuli presented are not interactive, thus do not change contingently with infants’ gaze direction. To ensure presentation times in the control procedure are similar in length to those in the intervention group, infants in the control group are matched infant-by-infant and visit-by-visit with participants in the ACT training. Thus, the cartoons displayed to a control child will follow the same schedule produced by the corresponding yoked child in the intervention group. In this way, the cartoons displayed to both groups will be the same, but the pivotal difference is that the display is generated contingently on infants’ gaze behaviour during treatment, and it is instead generated according to a pre-set schedule for those in the control group.

Whether the training or control procedure takes place in the dedicated room in the local charity or in the family’s home, we will use the same equipment consisting of a computer screen, a Tobii X-60 eye-tracker, Matlab scripts to control the visual display and record data provided by the eye-tracker, and a webcam to record the infants’ visual behaviour during the procedure. In both settings (local charity or family’s home), in order to avoid interference from light sources and to minimise distraction, the computer screen will be mounted inside a photographer’s white light tent, while the infant will sit on a parent’s lap on a chair inside this tent. To avoid distraction produced by noise, when visiting the family’s house, the researcher will ask the parent to identify times and dates whereby the house is quiet and will ask collaboration from parents in ensuring that possible sources of noise (e.g. appliances) are excluded.

### Outcome measures

#### Primary outcomes

- Recruitment as a percentage of the eligible families approached who agreed to take part in the study and were randomised, and retention, defined as the percentage of randomised participants for whom data are available at baseline and post-test.

#### Secondary outcomes

(b) Percentage of training/control sessions attended by infants, sessions completed by infants, duration of tasks completed during these sessions.
(c) Percentage and type of tasks for which data are available at post-test
(d) Quality of eye-tracker data collected during baseline and post-test assessments, such as the number of usable fragments and the degree of consistency in the reported position of gaze between recorded samples, and consistency of post-test outcomes with expected trends (e.g. general increase in disengagement abilities across study arms)

Furthermore, to assess the feasibility of the study, we also decided to collect feedback from participating parents using a short questionnaire and conducting a semi-structured interview. In the latter, parents are asked about difficulties and obstacles to taking part and remaining in the study, as well as their motivations in taking part, and their experience of the study.

### Baseline and post-test assessments

We devised a battery of tests and tools to be administered before and after the completion of training/control sessions: these provide information about the infants’ general cognitive and motor development, their attention, and their social cognition abilities. The presentation of tasks takes place in four pseudo-randomised sequences, counterbalanced between infants and across baseline and post-test within each infant. At baseline, parents provide socio-demographic information about them (e.g. educational attainment) and their child (e.g. birth weight), see Fig. 1.

#### General cognitive and sensory-motor development

We administered the Mullen Scales of Early Learning [43]. These scales provide individual scores on different domains (e.g. Expressive language; Fine motor abilities; etc.).

#### Computer-based measures of attention

These include widely used measures of sustained attention, visual recognition memory, disengagement, and information processing. These are described in more detail in Additional file 1.

#### Naturalistic attention tasks

We used the Orientation Task from the Lab-Tab [29, 50], as well as a semi-structured interaction between parent and infant. In the latter, four age-appropriate attractive toys are placed in front of the infant and the parent sits across the table. Parents are instructed to play with the infant as they “would normally at home”. The latter parent-infant play episodes last four minutes.

#### Social attention and cognition

We administered the Gaze Following, Object Spectacle, and Book Presentation task from the Early Social Communication Scales (ESCS) [44]. These tasks provide information on the infant’s ability to share attention with other people, and her communicative abilities.

#### Temperament

To assess temperamental measures, we administer the Task Orienting and the Attractive Toy Placed in a Box tasks from the Lab-Tab [29, 50]. The former also provides measures on infants’ focused attention, as well as temperamental measures relating to dimensions such as duration of orienting. The latter provides measures relating to behaviour regulation. Furthermore, parents are also asked to complete the very-short form of the Infant Behaviour Questionnaire (IBQ) [51].

### Sample size

The sample size has been determined using power calculations based on a confidence interval approach described by Cocks and Torgerson [17]. Based on a Cochrane review of interventions involving infancy outcomes of preterm infants [62], we estimated that the standard effect size for a cognitive training programme like ours is likely to be 0.40. The confidence interval approach prescribes a pilot sample size that can produce a one-sided 80% confidence interval upper limit which excludes the plausible a-priori effect size (0.40), assuming the training effect from the pilot was zero (no difference) or less (favouring the controls). In such a scenario, the sample size required would be 18 participants. We aim to recruit 20 infants in order to allow 10% drop out rate from the study.

### Randomisation

#### Sequence generation

The random allocation sequence was generated in two blocks (*n* = 10 each) using randomly generated numbers from the uniform distribution. These were generated using Stata 13 [63], and constraining the intervention/control ratio to be 1:1.

#### Allocation concealment mechanism

The main researcher conducting the trial received the allocation in a sealed opaque envelope when a new participant is due to commence testing. The researcher has been instructed to open the envelope only when the child finishes the baseline assessment.

#### Implementation

The sequence generation was produced by the first author and project PI. Participants are enrolled by the Experimenter 1 (training/control implementation). Experimenter 1 does not administer the baseline and post-test outcome measures, but during these, he attends to the cameras recording task administration. In doing so, he controls the camera remotely through a laptop while sitting outside the testing room.

#### Blinding

To ensure blinding, Experimenter 2 does not contribute to the administration of the training/control procedures, nor is he involved in coding or data analyses concerning training and control procedures. We inform parents that we will not tell them in which group their child is allocated. Parents are thus intended to remain blind to group allocation. Parents, however, keep their infant on their lap while the infant takes part in the training or control procedure, which raises the possibility that parents may identify whether the games are part of the training (i.e. they are responding in real time to the infant’s gaze) or the control procedure (i.e. games are not interactive). Based on experience from previous studies, we estimate it is unlikely parents will recognise the infant’s group allocation. Furthermore, while keeping the infant on their lap parents cannot monitor the infants’ gaze direction (i.e. they may not have key information to detect whether the games are interactive or not). However, at the end of the study, we ask parents to indicate whether they thought they had recognised to which study arm the child had been allocated, and in which study arm they thought their child was.

### Ethical approval

The study has been reviewed and approved by the Health and Social Care Research Ethics Committee A (HSC REC A), REC reference: 18/NI/0010; IRAS project ID: 237537.

### Governance and management

The study is sponsored by Queen’s University Belfast (QUB). QUB Research Governance Office audits research studies to make sure that they are being carried out in accordance with the highest standards of integrity. A trial steering group had been set up to meet at least four times during the running of the study. The group remits are to discuss progress of the trial, issues and problems arising, suggestions and solutions to these issues. The Trial Steering Group is made up of the study team members, as well as independent practitioners, and a parent representative.

Data are anonymised by assigning study IDs to each child and family taking part in the study. All questionnaires, coded data, and eye-tracking data are stored using these IDs. Anonymised data will be shared with researchers at QUB and University of East London (UEL) for research purposes. Video and audio records contain information that enables identification of the participants: for these reasons, these are stored on separate systems and are not going to be shared with researchers outside the sponsoring institution. In accordance with QUB Research Governance Framework, the information and the data collected will be stored for 5 years after study publication. The study was approved and started before the General Data and Privacy Regulation (GDPR) Act 2018 became effective. However, the procedures follow the spirit of the regulation (e.g. participants were informed about their right to delete information collected).

Audio recordings from parental interviews will be transcribed, and the original audio-recordings deleted once verbatim transcriptions have been carried out and checked. Researchers will delete from the transcripts any reference to sensitive information or information that may identify the parents or the children (e.g. parental place of employment). Parents agree in writing or in a recorded statement to the use of quotations from the interviews.

A distress protocol had been submitted and approved together with the Ethics application to the HSC REC. This protocol set clear criteria for interrupting singular sessions, as well as the study, if distress or other adverse circumstances involving the child, parents, or the family were reported by parents, or if distress and other adverse circumstances were noted by researchers during the study. The distress protocol also specified procedures to follow up or escalate concerns about participants’ health and wellbeing.

### Statistical analysis

Blinding of assessors was considered pivotal for this feasibility study. Statistical analyses are going to be conducted by research team members OP and SW, who will not be blind to trial arm. However, scripts of analyses and, whenever possible, data on which analyses are based will be made available to ensure transparency.

#### Primary outcomes

- Recruitment: a key outcome is how many parents have been contacted by gatekeepers (practitioners and the local charity), and the number of families that eventually agree to take part. Retention: we are going to consider the percentage of infants that complete at least 50% of the pre-test tasks and the percentage of infants that complete at least 50% of the post-test tasks to estimate the proportion of participants that dropped out. We also plan to carry out these comparisons between the intervention and control group. Finally, we are also going to investigate associations between drop out and infants’ and family characteristics such as infant’s gender, gestation age, admission to neonatal intensive care unit (NICU), family’s Social Economic Status (SES), e.g. differential rates of dropping out between males and females, or infants of different gestation age.

#### Secondary outcomes

(b) Percentage of training/control sessions attended and completed by infants: we will collect information on the number of sessions not attended (e.g. cancelled) by infants and examine differences across the intervention and control groups. We shall also examine other factors that may be predictive of non-attendance (e.g. infants’ gestational age). We also consider information regarding completion of sessions, defining a session as being completed if infants engaged without interruptions for the required time (240 s) with at least two tasks, irrespective of whether these were training or control tasks. Finally, we are going to investigate the number of tasks completed, defined as the display of the task for at least 240 s, the type of tasks completed (e.g. goal maintenance), the total duration of tasks administered to infants, and the average duration of tasks per session. We will test for differences in these outcomes by study group and by infants’ characteristics (e.g. gestational age).
(c) Data available at post-test: we will investigate the number and type of post-test tasks completed by infants and compare these between study groups and infants’ characteristics, e.g. gestational age and sex. We will also investigate differences in completion across tasks type in these categories: screen-based eye-tracking tasks; social attention tasks (ESCS tasks); Lab-Tab tasks; Mullen scales.
(d) Quality of eye-tracker data collected: we will calculate the duration (in seconds) of usable fragments of eye-tracking data obtained during randomly sampled tasks in the pre- and post-assessment, and during training/control sessions. These analyses will provide an estimate of the robustness of recording because ‘void’ fragments can signal issues capturing the infant’s eye gaze [75]. We will also investigate the consistency with which the gaze position reported by the eye tracker is consistent between samples, randomly selected from pre/post sessions and training/control sessions of participating infants. The latter measure provides an estimate of the precision of recording. These data will be compared with estimate from other studies involving typically developing infants, and we will also investigate differences across settings (e.g. charity vs. family home), as well associations between outcomes and infants’ characteristics. Consistency of post-test outcomes with expected trends: We will consider results in outcomes across the two groups and test if infants show expected trends in key tasks. In particular, we expect infants to display trends towards improved attention abilities in the screen-based eye-tracking tasks at post-test, as well as trends towards improved social attention and orienting in the ESCS social attention tasks, and the orienting task of the Lab-Tab. We will also compare differences across study groups in order to estimate effect size associated with the intervention.

#### Qualitative outcomes

Follow-up interviews with parents that have taken part in the study will be transcribed verbatim and analysed using thematic analysis to identify common themes related by participating parents. This analysis will be conducted by the study PI, while the RA will review a sample of the transcripts independently to identify themes. The two study team members will discuss disagreements and review their thematic analysis in light of this discussion.

## Discussion

### Importance of the study

The evidence of persisting intellectual and educational attainment deficits in children born VP [6, 15, 16] in spite of improvements in their care highlights the need for interventions that may enhance key foundational abilities at an early age. Over many countries, there is a recognition and acknowledgment of the role and cost-effectiveness of early interventions, and strategies for the provision of early interventions have been advocated by legislative bodies, e.g. the UK Parliament [35]. The ACT meets this need for early interventions, targets key skills that are related to the development of cognitive flexibility and Executive Functions, and does so at an age whereby attention control abilities are just emerging and may thus be particularly plastic and amenable to change [73]. The delivery of the ACT to infants born VP may be particularly apposite because VP infants are known to be at risk of deficits in control of attention [2, 23, 64], EFs [45], cognitive and intellectual abilities [15, 16]. Researchers recognise that there are wide individual differences in the developmental trajectories of VP infants, and many studies have also highlighted the resilience of many VP infants exposed to aversive events such as long stays in the NICU, and exposure to painful procedures [22, 24, 54, 70]. We want to emphasise that the ACT is not to be considered a remedial intervention for infants with recognised deficits, but rather a universal intervention that can help strengthen and reinforce key executive skills when they start to emerge. Nonetheless, infants born VP may particularly benefit from this intervention, as it may nourish abilities that some VP infants may find particularly challenging to master.

Before seeking funding for a full trial, it is important to ensure that the research processes and procedures of the ACT are safe, feasible, and acceptable to families of and infants born VP, and indicate whether or not a full trial is justified. This pilot trial is designed to address these issues.

### Determining the feasibility of a randomised controlled trial

Infants born VP are a relatively small sub-group within the overall infant population: in England and Wales, they represent approximately 1% of live births every year, but this still represents a large number of infants affected by VP birth, e.g. over 5500 in England and Wales [47]. These infants are often exposed to aversive events (e.g. long stay in the NICU) and may have complex needs, which affect the life and well-being of parents and families [3, 27].

The recruitment of this population in experimental studies and trials may thus present a series of challenges concerning identifying and contacting families affected by preterm birth, communicating in sensitive manners, challenges in participation and retention. This pilot study will help us identify optimum recruitment procedures, the likely time it will take to recruit a sufficiently powered sample size, what the barriers and facilitators of study participation might be, and whether the intervention is acceptable to participants and their families. We also aim to test the quality of information collected using eye-trackers and measures of infants’ engagement in the tasks, to ensure that equipment is suitable for recording the attention of VP infants, and that the procedures we plan to use are adequate and apposite for this population.

### Risks and benefits for participants

There will be small benefits for the families involved whether they receive the programme or not. They will experience five research visits, with a small monetary recompense, will observe the infant engaging in a number of naturalistic tasks (e.g. gaze following) that will help parents recognise their infant’s abilities, and will be able to talk about their child’s development: Experience in other studies suggests these opportunities are valued by families [68, 69].

The screening process by gatekeepers will also ensure that families of infants with complex or serious conditions will not be inconvenienced with requests to take part in the study. However, shall any concern about the health and safety of an infant or a family arise, this will be discussed with the collaborating consultant paediatricians. If paediatricians endorse these concerns, we will follow a protocol of actions, appropriate for the type of concern (e.g. discuss the concern with the family and suggest a consultation with a GP or a paediatrician). Information to this effect is included in the Patient Information Sheet.

### Dissemination

We will make public the results of the study via an open access journal publication, a final technical report and briefing for the funders of the study, and a plain English summary which we will send to all participants.

## Supplementary information

**Additional file 1.** Pre- and Post-test Tasks and Questionnaires.

**Additional file 2.** Training tasks.

## Data Availability

Not applicable: the manuscript does not contain any data.

## Abbreviations

ACT: Attention Control Training
ADHD: Attention deficit with hyperactivity disorder
EFs: Executive functions
ESCS: Early Social-Communication Scales
IBQ: Infant Behavior Questionnaire
NICU: Neonatal intensive care unit
SES: Social economic status
VP: Very preterm
