# Supplementary material for: Training attention control of very preterm infants: protocol for a feasibility study of the Attention Control Training (ACT)
Source: Pilot Feasibility Stud. 2020 Feb 10;6:17. doi: 10.1186/s40814-020-0556-9 (PMC7008548; doi:10.1186/s40814-020-0556-9)
Supplement: Supplementary file 1 — Additional file 1. Pre- and Post-test Tasks and Questionnaires. [file 40814_2020_556_MOESM1_ESM.docx]

## Additional file 1: Pre- and Post-test Tasks and Questionnaires

Screen-based attention tasks (Pre- and post- test)

These tasks will be administered to all infants before the training/control procedure in Visit #1, and one week after the end of the training/control procedure in Visit #5. The tasks assess different aspects of attention and cognitive control compared to those targeted by the training, **thus testing transfer of effects**. The order of the tasks will be a pseudo-randomised order. The administration of these tasks is expected to last approximately 20 minutes, but each task has built-in criteria for terminating the task based on infants’ engagement, and therefore total time of presentation may vary. In order to maintain infants’ engagement, different short video-clips will be presented between tasks to attract infants’ attention on the screen. Infants’ direction of gaze during these tasks will be recorded using the eye tracker.

The tasks are described in more details in what follows, providing examples of stimuli (taken from Wass et al., 2017):

| **Sustained attention.**  A still image of a child’s face on a white background will be presented in silence. The RA watching a live video feed of the child from behind a curtain, coded whether the child is looking at the screen using a key press. When the child looks away for 1 s or more, this marked the end of the trial. The  same image will then be presented again until the child completes two consecutive looks at less than 50% of the longest look displayed (Colombo & Mitchell,  2009). If the child has not met the habituation criteria within 120 s of accumulated looking time or within 12 trials, the trial is aborted and the block excluded. Measures will include peak look, calculated as the duration of the single longest unbroken look toward each stimulus, and looks to criterion, calculated as the number of looks required to  fulfil the habituation criterion (Colombo & Mitchell, 2009). |    |
| --- | --- |
| **Visual Paired-Comparison (Visual recognition Memory)**  The same familiar image from the previous task will be presented concurrently with a novel image over three trials. The images will be presented in one Left-Right order for 8,000 ms, successively swapping the order for the subsequent 8,000 ms. Proportion looking to the novel target will be calculated as time spent viewing the unfamiliar target, divided by the time spent viewing both the familiar and the unfamiliar target combined. |  |
| **Gap–overlap (Disengagement of Attention task).**  This task will be presented in three blocks. The first two blocks will last 20 trials, while the third will continue until 12 usable trials per condition have been collected, or 80 trials have been presented, or the infant becomes inattentive.  After fixating a central target (CT), following a variable Inter-Stimulus Interval (ISI) a lateral target (LT, a cartoon cloud) will appear to either the left or right; when the infant fixates the LT, a brief audio-visual display is presented on the screen. Three conditions will be used:  **Gap** – CT will disappear 200 ms *before* LT appears;  **Baseline** – CT disappears *concurrently* with LT appearance; **Overlap** – CT remains on screen *with* LT appearance.  The order of trials will be randomised between conditions. The key variable of interest is the reaction time (RT), namely the time elapsed between LT appearance and the reported position of gaze leaving the central fixation area. Reaction times less than 100 and greater than 2000 ms will be excluded. Average reaction times will be calculated by first averaging the reaction times obtained across the three separate conditions, and then combining the log transformed averages to create a final average. Following Elsabbagh et al. (2009), disengagement latencies will be calculated as the participant's average reaction time in the overlap condition subtracted from their average reaction time in the baseline condition. |  |
| **Information Density Preference**  Six blocks are presented at different times during the testing session (3 conditions x 2 blocks for each condition). Each block lasts 100 seconds, and features 4 images that are presented in rotation. In condition 1, ‘slow’, each picture is presented once, for 25 seconds, during the block. In condition 2, ‘medium’, each picture is presented for 10 seconds. After each picture has been presented once, the sequence of 4 pictures repeats. In condition 3, ‘fast’, each picture is presented for 1 second. After each picture has been presented once, the sequence of 4 pictures repeats.  The dependent variable is look duration, measured by the researcher manually coding the infants looks to and away from the screen using a key press. | 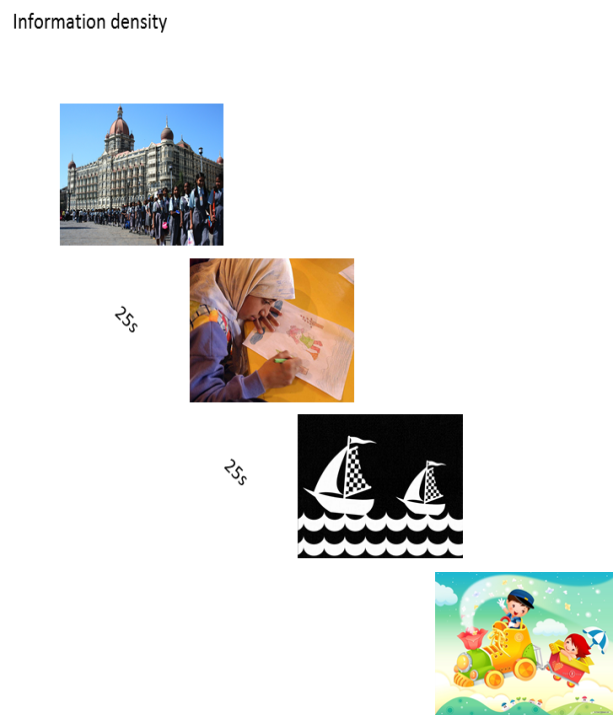 |

Early Social Communication Scales (Pre- and Post-test)

Tasks from these scales will be administered to **assess infants’ social attention and social cognitive skills** during controlled observations with an experimenter. The tasks will be administered while the infant sits on the mother’s lap across a table, with an experimenter sitting opposite the infant. The tasks will be video-recorded to allow further analyses. The order of presentation of the tasks will be pseudo-randomised.

*Object spectacle task*: Three wind-up mechanical toys and three hand-held toys (e.g. squeeze toy) are displayed on a table. The researcher will activate the toys in front of the infant, but ensuring it is out of reach. The researcher will remain silent, but if the child initiates a communicative bid (e.g. alternates her gaze between researcher and toy), the researcher will provide a natural but brief response (e.g. smiles and nods). If the infant tries to obtain the toy, the experimenter will move the toy within the infants’ reach. If the toy ceases but the child has not made a bid for it, the experimenter will place the toy within the infant’s reach and allow her to pay with it for 10 sec. Each toy is activated and presented in turn three times in a row. This task measures initiating joint attention and initiating behavioural requests.

*Gaze following task*: The experimenter will attract the infants’ attention. When the infant is watching the experimenter’s face, the experimenter turns her head and points to one of four posters located either on the left side, or left behind the infant, or on the rift side, or right behind the infant. While looking and pointing at the poster, the experimenter will call the child’s name three times, each time more emphatically. The experimenter’s turn and pointing to the poster will last at least 6 seconds. After this, the experimenter will attract the child’s attention back and make a comment on the poster (e.g. “Did you see Pooh?). The experimenter will initiate another trial when the infant is looking at the experimenter’s face. The experimenter will turn and point to each one of the four posters. The order of direction of turns will be pseudo-randomised. The task assesses infants’ ability to follow another’s person gaze towards a target. The task will be repeated two times during the session.

*Book presentation task*: A picture book will be presented on the table within infant’s reach. The experimenter will ask the child “What do you see?” and will allow the child 20 seconds to examine the book. If the child points to pictures in the book, the experimenter will respond briefly (e.g. “Yes, I see”; “That’s nice”). After the initial 20 seconds, the experiment will start pointing to pictures in the book, regardless of whether the infant had spontaneously pointed at them or not. Each pointing gesture will be maintained for at least 3 seconds, avoiding tapping or touching the picture while pointing. The experimenter will also call the child’s name while pointing to pictures on the left side of the book, then point to a new picture on the right side on the same page. After these two pointing gestures, the experimenter will repeat this procedure two times, turning onto a new page in the book. This task assesses infants’ ability to initiate joint attention bids (e.g. spontaneously point to pictures, alternating gaze between experimenter and pictures) and their ability to respond to joint attention (follow the experimenter’s pointing gesture).

LabTAB (Pre- and Post-test)

Two tasks from the LabTAB will be administered to all infants during the post-test in visit #5. Similarly to the ESCS, these will be administered while the infant is sitting on her mother’s lap in front of a table. The tasks will be video-recorded to allow further analyses of infants’ performance. **These tasks provide naturalistic measures of attention and social attention skills.**

*Task orientation task*: The experimenter will instruct the infant’s parent/caregiver saying : “In this task we are interested to see how children manipulate a set of blocks. Please allow [name of child] to play with the blocks on his/her own. If [name of child] seeks your involvement you may say ‘yes, you play with it’. If the blocks have fallen on the floor or out of reach, and you can reach them, please put them back on the table in front of your child”. The experimenter will then present a set of wooden blocks and say “Here are some blocks for you to play with”, then will address the parent saying: “I’ll be 3 minutes”, and will stay behind the infant and the parent. The task will provide an assessment of infants’ orienting and focused attention skills in a controlled naturalistic setting.

*Attractive toy placed in box:* The researcher will sit 90 degrees from the infant holding some papers to read. A plastic transparent box with a removable lid and an attractive toy (e.g. a colourful ball) will be used in this task. The experimenter will instruct the parent saying: “In this task we are interested to see how children react when the toy they are playing with is taken away for 30 seconds at a time. [name of child] is likely to show some signs of frustration during this task, but I will only take the toy away on three occasions and he/she will have a chance to play with it in between. Please do not help him/her retrieve the toy. If he/she seeks your involvement, you may say “yes, the toy is in the box’. If he/she seeks your comfort, by all means comfort him/her. To avoid distraction, I will not talk to [name of child] during this task. If you feel uncomfortable continuing with this task at any point, please tell me and I will stop the task immediately”. The experimenter will then display the toy and show the infant how it can be played with, and then place it within the child’s reach. After the child has played with it for 30 seconds, the experimenter will take the toy and place it in the box, closing the lid and leaving it there for 30 seconds. During the latter period, the experimenter will pretend to be reading from her papers. The toy will be then returned to the infant, and the procedure repeated 2 more times. The task assesses infants’ orienting, social referencing, and behaviour control.

Mullen Scales of Early Learning (Pre- and Post-test)

The Mullen scales represent a validated assessment of cognitive and motor abilities suitable for infants from birth to 68 months of age. The assessment involves presenting age-appropriate tasks to the infant (e.g. invite the infant to roll a ball) using test material similar to ordinary toys (e.g. toy cars, balls, etc.). The scales provide standardised scores in different areas: gross and fine motor abilities, visual reception, expressive and receptive language. Furthermore, these scores can be used to calculate an Early Learning composite score, thus providing a validated and standardised test of general cognitive abilities.

Parent-infant free play (Pre- and Post-test)

The parent (or caregiver) that accompanies the infant to the session will be also asked to play with the infant for 5 minutes, while we will video-record this free play session. We will provide standard toys in the room (e.g. wooden blocks), but will not instruct parents to use them. The instructions we will provide are: “We now want to see how (baby’s name) behaves in typical game situations. Therefore, can you please play with (baby’s name) as you would do at home if you had some time together? There are some toys in this corner that you can use if you want. We will video-record you and (baby’s name) for five minutes, but we won’t interfere. If (baby’s name) becomes tired or needs a break, just ask us to stop.”

The recording from these sessions will be used to investigate infants’ ability to focus attention on objects and on the parent, as well as abilities to coordinate attention between objects and the parent, and other social attention skills (e.g. direct the parent’s attention to objects).

Infant Behavior Questionnaire (Pre- and Post-test)

We will ask parents to complete the very short form of this questionnaire before the first session and then at the conclusion of the study. The questionnaire asks a series of questions concerning the infants’ behaviour as observed by parents in daily situations. The questionnaire provides a validated assessment of infants’ temperamental traits. In particular, we are interested in traits that are related to attention and behaviour control (e.g. activity levels of the infant, distress to limitation, or duration of orienting).
