# Supplementary material for: Training attention control of very preterm infants: protocol for a feasibility study of the Attention Control Training (ACT)
Source: Pilot Feasibility Stud. 2020 Feb 10;6:17. doi: 10.1186/s40814-020-0556-9 (PMC7008548; doi:10.1186/s40814-020-0556-9)
Supplement: Supplementary file 2 — Additional file 2. Training tasks. [file 40814_2020_556_MOESM2_ESM.docx]

## Additional file 2: Training tasks

The training tasks described below and cognitively similar variations of them will be administered in each training session (visits 1 to 4) until the infant stops engaging with the task. The tasks will be presented in pseudo-randomised order, ensuring the order is counterbalanced within and between participants.

| **Butterfly**.  A target (a butterfly ) will be presented on the screen. When the child fixates the butterfly, the butterfly flies across the screen, while distractors (a house, a tree, clouds) scroll in the opposite direction. When the child looks to any of the distractors they disappear and only the butterfly, now static, remains on screen. On re-fixating the butterfly it re-commences moving and the distractors re-appear and continue scrolling. The salience of the distractors will change adaptively, including faster, larger and more densely packed objects. This task rewards a child for maintaining her  fixation on one target, while suppressing the pre-potent response to look towards moving distractors in the periphery. |  |
| --- | --- |
| **Fly me.**  Similar to butterfly: A pink shape appears on the screen: when the child fixates it, face-like features appear on the shape (eyes and mouth) and it moves across the screen. Distractors appear moving in different directions. When the child looks away from the target character, it stops and its face-like features fade out while at the same time the distractors fade out from the screen. The target character resumes moving its face-like features reappear when the child fixates it again. The distractors change adaptively. | 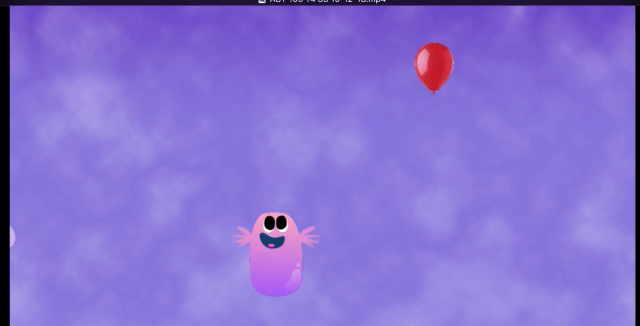 |
| **Stars**.  One of five possible targets (cartoon characters in brightly coloured stars) will be presented on screen together with eight distractors (smaller stars, planets, clouds). If the infant looks to the target within 3000 ms, she receives an animation as a reward. The target changes from trial to trial. The salience of the distractors changes adaptively. At lower difficulty levels, the eight distractors will be smaller, static, and identical to each other and dissimilar from the targets. At higher difficulty levels, they will be more varied, moving, brightly coloured, and similar to the targets. |  |
| **Windows**.  When the infant fixates the target (an animal in a window), an animation shows the target disappearing into one of several windows, which are then covered with curtains. A fixation target (a flower) appears elsewhere on the  screen and rotates when the infant looks at it. After a delay period, the fixation target disappears. If the infant looks back to the window behind which the target had disappeared, she receives an animation as a reward. The number of windows, the salience of the distractors, and the length of the delay changes adaptively. This task trains visuospatial working memory and requires acting on stored information about objects embedded in complex scenes. |  |
| **Puzzle memory**.  Similar to Windows: a character moves into one out of two (or more) squares, then disappears behind a screen. The screen then becomes black and a central stimulus appears (a ball moving from top midline to bottom) in order to attract infants’ attention. After this, the two squares reappear covered by the screen. After the infant looks at the square where this character had been, the character is revealed and stays inside the square. A new character appears, moves into the other square, the screen becomes black and the central stimulus (moving ball) is displayed. After this, the two squares reappear: the first character is still visible inside the square, while the other square is covered by the screen. When the infant looks at the square where the novel character was, the character pops out producing a sound. | 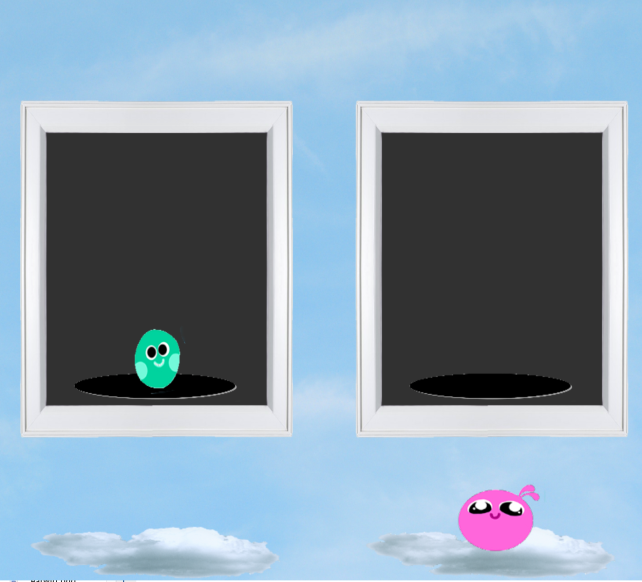 |
| **Tausendfuss**  Similar to puzzle memory. An animated character appears inside a square, moves and makes a sound, then disappears behind a screen that covers both squares. A central stimulus than appears in the top midline of the screen (a moving spiral). If the infant looks back at the square where the animated character had appeared, the character pops out and makes a noise. Following this, when the infant looks at the other empty square, a new animated character appears. This is also covered by a screen, while the first character remains visible in the other square. A central stimulus appears again. After this, the second novel character pops out of the covered square if the infant looks at it for a pre-defined amount of time. | 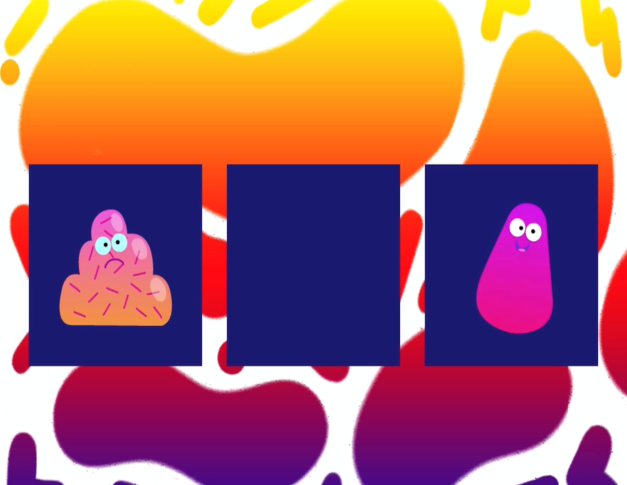 |
| **Three Little Maids.**  A character appears on the screen, then is covered by one of two (or more) pots that move into the screen. After this, a central fixation target appears on the screen to attract the infants’ attention to midline (a moving cartoon spider). After the central fixation stimulus disappears, the child is rewarded by an animation if she looks back (within a pre-set time limit) to the pot in the location where the original character had been. The task changes adaptively by increasing the number of pots on the screen from which to select the target. | 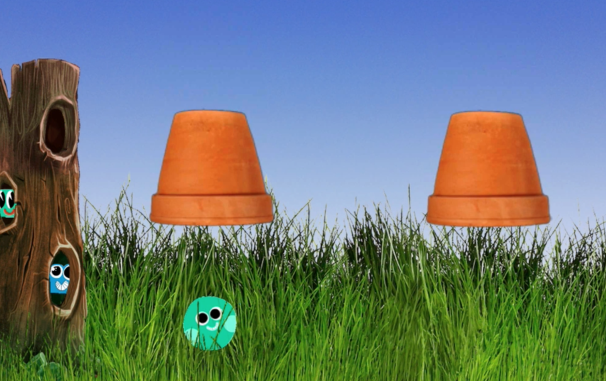 |
| **Suspects**.  One of two possible targets (either an elephant or a chicken) will be presented with one or more distractor items of the same size. When the infant looks at the target within a time limit, she receives an animation as a reward. The same target is then re-presented with other distractor(s). The number of distractors varies adaptively with performance; at higher performance levels, more distractors are presented. Between blocks of 12 trials, the target changes: where previously the infant had received a reward for looking to the elephant, she will be rewarded for looking to the chicken. At higher difficulty levels, the target from the previous block is presented concurrently with the target from the current block (a conflict trial); at lower difficulty levels, only novel distractors are presented (non-conflict). This task targets attention shifting and flexible search for changing targets, whilst ignoring distractors. |  |
| **Disengagement.**  A central moving stimulus appears on the screen (a colourful flower), and after that, two characters appear. If the infant watches at one of the two chosen at random by the computer script, a rewarding animation is displayed. In successive trials, the infant receives the animation every time she looks at the pre-set target character. The number of distractors increases adaptively. | 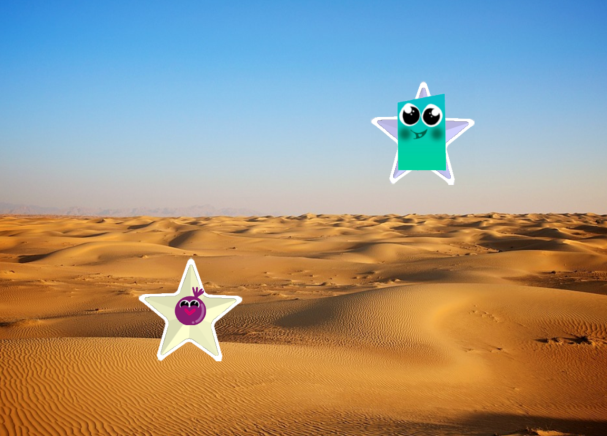 |
